# Raise two effects with one scene: scene contexts have two separate effects in visual working memory of target faces

**DOI:** 10.3389/fpsyg.2014.00400

**Published:** 2014-05-08

**Authors:** Azumi Tanabe-Ishibashi, Takashi Ikeda, Naoyuki Osaka

**Affiliations:** ^1^Department of Psychology, Graduate School of Letters, Kyoto UniversityKyoto, Japan; ^2^Department of Mind and Brain Science, Graduate School of Human Science, Osaka UniversityMinoh, Japan; ^3^Department of Adaptive Machine Systems, Graduate School of Engineering, Osaka UniversitySuita, Japan

**Keywords:** visual working memory, context effects, face memory, scene recognition, semantics

## Abstract

Many people have experienced the inability to recognize a familiar face in a changed context, a phenomenon known as the “butcher-on-the-bus” effect. Whether this context effect is a facilitation of memory by old contexts or a disturbance of memory by novel contexts is of great debate. Here, we investigated how two types of contextual information associated with target faces influence the recognition performance of the faces using meaningful (scene) or meaningless (scrambled scene) backgrounds. The results showed two different effects of contexts: (1) disturbance on face recognition by changes of scene backgrounds and (2) weak facilitation of face recognition by the re-presentation of the same backgrounds, be it scene or scrambled. The results indicate that the facilitation and disturbance of context effects are actually caused by two different subcomponents of the background information: semantic information available from scene backgrounds and visual array information commonly included in a scene and its scrambled picture. This view suggests visual working memory system can control such context information, so that it switches the way to deal with the contexts information; inhibiting it as a distracter or activating it as a cue for recognizing the current target.

## INTRODUCTION

Many people have had the experiences of failing to recognize a person whom they were sure that they had seen before. Mandler (1980) called such experiences the “butcher-on-the-bus” phenomena, based on his experience of seeing a man on a bus whom he only recognized later when seeing him as the butcher at his favorite supermarket. Despite its frequency, little is known about why this phenomenon occurs. Hayes et al. (2007, 2009) assume that the memory representation of the object is associated with its scene context, which explains decreased memory performance for the object when it is presented with another scene. Based on this account, they renamed the phenomenon as the “Context Shift Decrement” in their series of neuroscientific studies. Gruppuso et al. (2007) posited that the strength of the association between a target and its context in long-term memory (LTM) determines whether the “butcher-on-the-bus” effect occurs, that is, if the association is not strong enough, the target information is insufficiently recollected. On the other hand, despite the short duration of memory retention, some researchers have reported scene context effects in short-term memory (STM) and working memory (Hollingworth, 2006, 2007; Tanabe and Osaka, 2009; Nakashima and Yokosawa, 2011), which suggests the cause of the “butcher-on-the-bus” effect might be generated in a short-time process before the consolidation of LTM. In fact, Fitzgerald et al. (2011) reported that face processing in STM affected the confidence of LTM. Therefore, the context effect on memory should be examined in a paradigm of STM or working memory.

Furthermore, it is proposed that short-term storages in the working memory model are controlled by the central executive (Baddeley, 1986, 2000). According to Miyake et al. (2000), the executive function consists of three components: shifting of mental sets for the task (“shifting”), updating and monitoring of task-relevant information (“updating”) and inhibition of task-irrelevant information (“inhibition”). In particular, the “inhibition” component is regarded as an important function for visual working memory in rich contexts, because the information from the surroundings needs selection of the information to remember and control of the rest of information to prevent remembering. Our previous study revealed an ability for inhibition of task-irrelevant information from contexts correlated with visual working memory capacity (Tanabe and Osaka, 2009). That is, people who are not able to inhibit task-irrelevant information failed to recognize the information that they were instructed to memorize. We found that they were sometimes distracted by task-irrelevant information from contexts that were presented at encoding and falsely recognized the irrelevant context information as the memory target. In the previous study, we concluded that inhibition, a part of the executive function, should play an important role in visual memory with contexts and that, in particular, the executive function should control context information whenever people need to memorize a certain piece of information from it. Considering this, in the current study, we examined context effects on working memory, such as the “butcher-on-the-bus” effect, as well as how the context information was controlled.

Context effects may be explained by the encoding specificity principle (Tulving and Thomson, 1973). This principle proposes participants remember better when targets at test are presented in the same contexts as those at encoding. However, the influence of context on memory remains controversial (for reviews, see Nairne, 2002). For instance, some studies have suggested that the common operations at encoding and test facilitate recognition performance in a within-groups design, but disturb recognition performance in a between-groups design (Mulligan and Lozito, 2006; Dewhurst and Brandt, 2007; Dewhurst and Knott, 2010). Similarly, some studies on STM or working memory have indicated that contexts facilitate the target memory, while others found they disturb it. For example, Hollingworth (2006) reported that the memory performance of objects in scenes was higher when they were presented within the same scenes as those shown at the encoding phases than when target objects were presented in isolation, concluding that the association between an object and a scene facilitates object memory. Conversely, Liu and Jiang (2005) reported that the performance of object recognition with scene contexts was lower than the performance without scene contexts. From their results, they surmised that the scene contexts increase the visual complexity of the display and therefore disturb object memory since working memory is strictly capacity-limited. Both of these two studies implied that the memory performance is higher when the context in the retrieval phase is same as that in encoding phase. However, it remains unclear whether the same context as the encoding phase facilitates memory of the target, or a change to the context disturbs it. To examine whether scene contexts facilitate or disturb object memory, we made a comparison between the context effects with the same scenes, with different scenes and without scenes. If scene contexts facilitate memory, the performance with the same contexts would be higher than that with different contexts or without scenes. In contrast, if processing scene contexts disturb memory, the memory performance without scenes would be higher than different scene contexts.

Another question regarding the context effect is what information from scenes influences object memory. In Paivio’s well-known dual-coding theory, which was proposed as a model of LTM, there exist two independent but partially interconnected processes of memory representation, verbal (semantic) and non-verbal (perceptual) processes (Paivio, 1971). According to Paivio and Csapo (1973), nameable objects can be encoded both as verbal and visual representations, thus the performance in object recognition is generally higher when the items are presented as pictures than when they are presented as concrete nouns. Following the dual-coding theory, scene images are encoded as semantic and perceptual representation, therefore it is possible that semantic and perceptual information of scenes have different influences on the memory of targets. Velisavljeviæ and Elder (2008a,b) compared recognition performances of a fragment of a scene image with that of a scrambled image and revealed lower performances when the fragment was presented with the scrambled scene than with the original scene. Since it is harder to access the semantic information from a scrambled image than the original image, they suggested that semantic information extracted from a scene image improves recognition of a part of the scene. Inoue and Takeda (2012) reported scrambling scene reduces memory performance of the attended abject in the scene, although this effect was not observable when the object was not attended. Cowan (2001) argues capacity-limited attention constrains working memory capacity. Therefore, semantic information of a whole scene will affect visual working memory performance when participants deploy their attention to a part of the scene. Meanwhile, some researchers have reported that matching perceptual features of backgrounds between the encoding and test phases enhances performance of LTM recognition (Graf and Ryan, 1990; Reder et al., 2002; Reingold, 2002; Gardiner et al., 2006). Sun and Gordon (2009, 2010) set a target object surrounded by other objects and investigated whether an array of objects functions as a context in visual STM. They found perceptual changes of surrounding objects decrease the memory performance of the target object. It is possible that perceptual information of a scene background have a context effect on memory of the object in the scene. In sum, the simultaneous contributions of semantic or visual array information of scenes are not completely differentiated in the past studies, and, hence, the separable effects of those two types of information on working memory are not fully understood.

Taking into account findings of working memory and context effects, it is conceivable that working memory function could control task-irrelevant information, such as contexts. However it remains unclear what cognitive processes on semantic and/or perceptual visual array information influence object memory. Therefore the purpose of the current study is to answer the two questions; (1) whether contexts facilitate or disturb memory, and (2) whether semantic or visual array information contributes to context effects. To answer these questions, we compared the context effects between meaningful scene backgrounds and meaningless visual backgrounds to disentangle the influences of semantic and visual array information. We conducted an experiment of the “butcher-on-the-bus” effect on working memory with a delayed-match-sampling recognition task for faces and measured the memory performance between three conditions: tests with the same backgrounds as the encoding phases, tests with different backgrounds from those in the encoding phases and tests without backgrounds. To examine the effect of visual array information, we used scrambled scene backgrounds that held visual array information but were void of the semantic components. Comparing the results between the scene backgrounds and the scrambled backgrounds, we discussed how the natural scene backgrounds cause the contextual effect in everyday memory, such as the “butcher-on-the-bus” effect.

## MATERIALS AND METHODS

### STIMULI

We used 441 achromatic composite photographs as the stimuli. Original scene photographs were collected from the database of the Computational Vision Group at the California Institute of Technology. There were four categories of photographs: “inside-city,” “living-room,” “forest,” and “coast.” Original face photographs were collected from the databases of the Psychological Image Collection at the University of Stirling and from the Computer Vision Research Project at the University of Essex. The faces consisted of 126 Caucasian males and 63 Caucasian females. Asian participants might have difficulty remembering Caucasian faces, but the influence of faces from other racial backgrounds would prevent a ceiling effect. Such “the other race effect” (for review, see Meissner and Brigham, 2001; Sporer, 2001) should not have an effect on the difference of conditions because the images in all conditions were from the same racial background. The appearance of males and females was counterbalanced. Scrambled images as backgrounds were created by dividing the scene photographs into a 30 × 30 grid of tiles and randomly reassigning the tile positions by Visual Basic 6.0. The Scrambled images were made from the same photographs as the ones used in scene backgrounds. Using GIMP for Windows 2.6 (http://www.gimp.org/), the faces were superimposed on the background images and the stimuli were edited to a uniform size (200 × 200 pixels). The size of each face was modified to be about one third of the stimulus.

### PARTICIPANTS

Forty Asian students (17 males and 23 females; mean age, 23.0; SD, 2.55) at Kyoto University participated in the experiment as volunteers. All participants provided informed consent to participate in the current experiment. They reported normal or corrected-to-normal vision. The participants were pseudo-randomly assigned into two groups: half to the task with scene backgrounds, and the other half to the task with scrambled backgrounds.

### PROCEDURES

**FIGURE 1:**
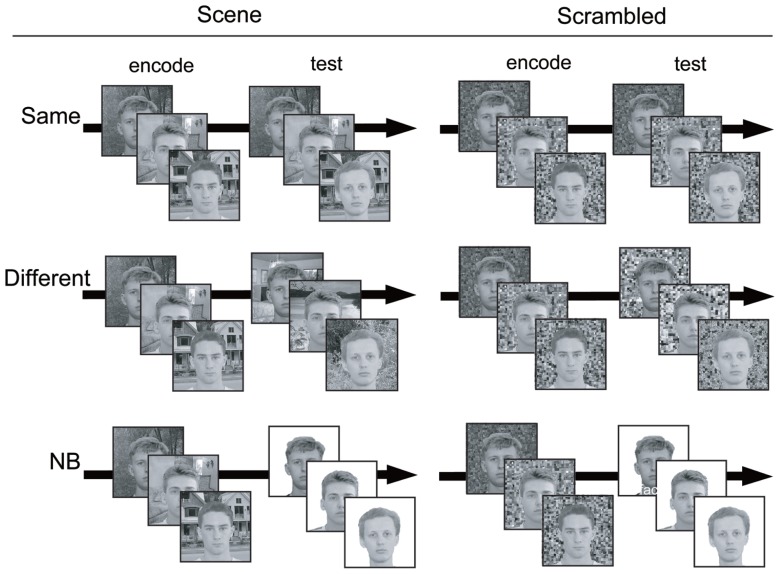
**Examples of three conditions in the current experiment.** In actual procedure, the fixation point was presented after encoding images.

At the beginning of a trial, participants were instructed to memorize faces in scene images. One trial consisted of three images and each image was presented for 1 s. After an 8-second delay with fixation, a probe image for each encoded image was presented and serial recognition was performed by key press on the keyboard. Three recognition probes were presented in the order corresponding to the presentation sequence of the encoding images. Participants were instructed to press the “1” key when they saw the encoded face and to press “3” key when they saw a novel face as accurately and quickly as possible. To avoid a wrong key press, we set the key between the two response keys as invalid. Half of all probes in each condition of the experiment were novel faces. The frequency and the serial order of the novel probes in each trial were counterbalanced. To examine the influence of the context information of the backgrounds on recognition, we established three conditions: “Same,” “Different,” and “NB” (see **Figure 1**). The details of each condition are as follows:

Same: face recognition with the SAME background image as encoding
Different: face recognition with a DIFFERENT background image from encoding. The background image was from a different category than was presented at encoding.
NB: face recognition with NO BACKGROUND image

In each condition, 14 trials were performed. The trials in each condition were randomly presented. Before the experiment began, participants completed a practice session of six trials. The experiment was performed on a Windows XP personal computer using Presentation 14.2 (Neurobehavioral Systems).

### DATA ANALYSES

To compare the performance of recognition, we calculated A’ as an index for memory performance for each condition and measured reaction times (RT) with millisecond time resolution of the trials that participants answered correctly. A’ is an index for recognition memory based on the signal detection theory similar to d’ (Snodgrass and Corwin, 1988), but it can be applied when hit rates are 100% and when false alarm rates are 0%. We used A’ as the index for accuracy because some participants showed such high performances. Furthermore, we examined hit rates and false alarm rates separately to investigate to which process(es) of memory the contexts contribute. If the same context as encoding contributes to familiarity but not to accuracy of the memory, both hit rates and false alarm rates should be higher for the Same condition compared to the other two conditions. Therefore, similar memory performance would be predicted regardless of the context conditions. In fact, Hockley (2008) observed in LTM experiments that both hit rates and false alarm rates are higher when recognizing targets in old contexts than in new contexts. However, if the context contributes to the accuracy of the memory, false alarm rates should be indistinguishable in any of the three conditions.

## RESULTS

**FIGURE 2:**
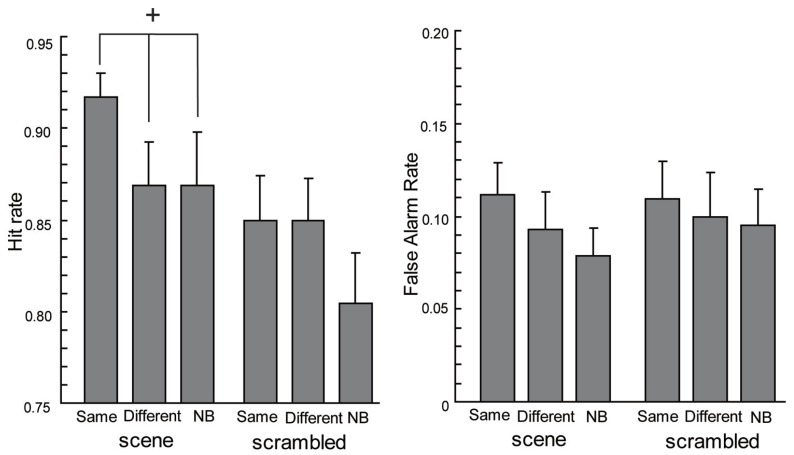
**The hit rates and false alarm rates of the current experiment.** Error bars are standard errors of means. The cross means a significant tendency (*p* < 0.1).

To investigate the context effect, we analyzed hit rates and false alarm rates separately. Regarding hit rates (see the left side of **Figure 2**) with scene backgrounds, one-way ANOVA showed the main effect tended to be significant [*F*(2,38) = 2.92, *p* = 0.07]. A *post hoc* comparison of the context conditions by Ryan’s (1959) method, showed the Same condition tended to have higher hit rates than the Different condition [*t*(38) = 2.09, *p* = 0.04] and the NB condition [*t*(38) = 2.09, *p* = 0.04]. There was no significant difference between the hit rates in the Different and the NB conditions [*t*(38) = 0, *p* = 1.00]. On the other hand, ANOVA for the hit rates with scrambled backgrounds did not show a significant effect [*F*(2,38) = 1.82, *p* > 0.1]. Moreover, ANOVAs of the false alarm rates (see the right side of **Figure 2**) showed no significant effects [the main effect under the scene condition, *F*(2,38) = 1.52, *p* > 0.1; the main effect under the scrambled condition, *F*(2,38) = 0.25, *p* > 0.1]. These results are in agreement with context effects on recognition observed in LTM (Rutherford, 2004; Isarida et al., 2005).

**FIGURE 3:**
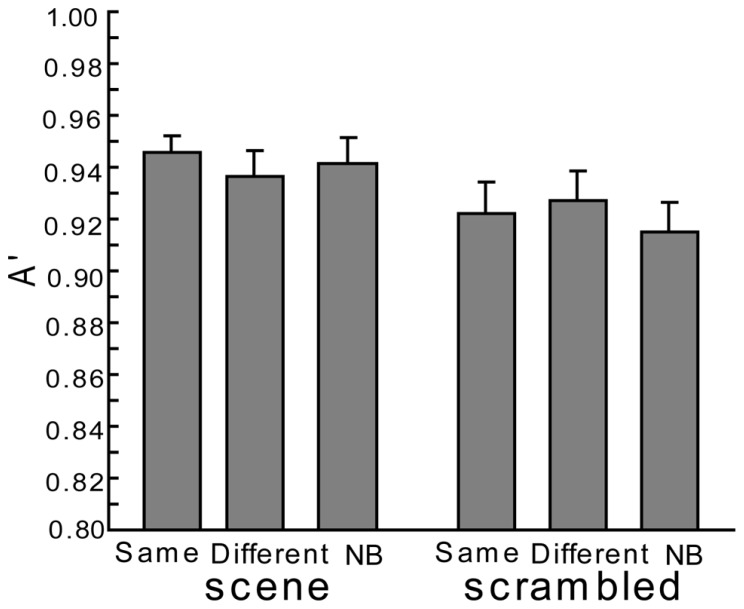
**The accuracy (A’) of the current experiment.** Error bars are standard errors of means.

In addition, no significant differences were seen for A’ with both scene and scrambled backgrounds [see **Figure 3**, the main effect under the scene condition: *F*(2,38) = 0.43, *p* > 0.1; the main effect under the scrambled condition: *F*(2,38) = 0.61, *p* > 0.1]. As **Figure 2** and the former paragraph describes, both hit rates and false alarm rates (though not statistically significant in the current experiment) in the Same condition tended to be moderately high so that the scene context would fail to facilitate the accuracy of recognition in comparison to the other conditions. The accuracy of the recognition in each condition was high; at most, there were only slight differences in performance for each type of background.

**FIGURE 4:**
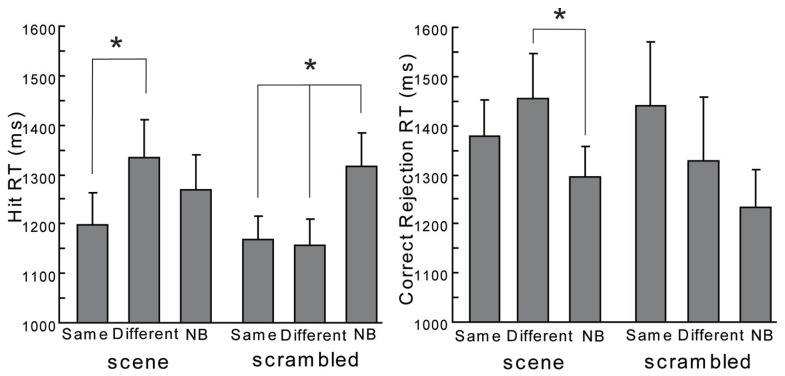
**The hit RT and the correct rejection RT in the current experiment.** Error bars are standard errors of means. The asterisk means a significant difference (*p* < 0.05).

In contrast, a one-way ANOVA for RT of hit trials (hit RT) did show significant differences both with scene and scrambled backgrounds [see the left side of **Figure 4**; the main effect under the scene condition: *F*(2,38) = 3.98, *p* < 0.05; the main effect under the scrambled condition: *F*(2,38) = 5.97, *p* < 0.01]. Multiple comparisons by Ryan’s (1959) method, demonstrated that for the scene backgrounds RT under the Different condition was longer than that under the Same [*t*(38) = 2.82, *p* < 0.05]. The other comparisons did not show significant differences in the scene backgrounds [Different vs. NB: *t*(38) = 1.31, *p* > 0.1; Same vs. NB: *t*(38) = 1.51, *p* > 0.1]. The hit RT under Different condition with scrambled backgrounds was longer than that under Same condition [*t*(38) = 2.82, *p* < 0.05] or NB condition [*t*(38) = 3.12, *p* < 0.05]. There was no significant difference in the hit RT between the Different condition and the Same condition with scrambled backgrounds [*t*(38) = 0.27, *p* > 0.1]. Furthermore, one-way ANOVAs for RT of correct rejection trials (correct rejection RT, see the right side of **Figure 4**) with scene backgrounds revealed a significant difference [*F*(2,38) = 4.46, *p* < 0.05] but not with scrambled backgrounds [*F*(2,38) = 1.30, *p* > 0.1]. Multiple comparisons by Ryan’s (1959) method, showed RT under the Different condition with scenes was longer than that under NB condition [*t*(38) = 2.99, *p* < 0.05]. The other comparisons did not show significant differences in the scene backgrounds conditions [Different vs. Same: *t*(38) = 1.40, *p* > 0.1; Same vs. NB: *t*(38) = 1.58, *p* > 0.1]. To summarize, the same scenes tended to enhance the hit rates only modestly and different scenes delayed the responses. Regarding the scrambled backgrounds, the absence of backgrounds at retrieval made the responses slower. The context effects were produced in different ways between the scene and scrambled backgrounds.

## DISCUSSION

Our experiment aimed to answer (1) whether facilitation of target memory by identical contexts in the encoding and test phases or disturbance of target memory by different contexts in the two phases, best explains the encoding specificity principle for target recognition in working memory; and (2) whether semantic or visual array information from the backgrounds is critical for the encoding specificity principle. We found that the changes in scene backgrounds and the absence of scrambled backgrounds make RT longer, and the same scene backgrounds increased the hit rates modestly, but not the false alarm rates. From these results, we drew two conclusions.

One conclusion from the current experiment is that the semantic information of contexts may facilitate retrieval of target objects as a memory cue. As mentioned above, the same scene backgrounds tended to enhance the hit rate more than the different scenes or white backgrounds, but the scrambled backgrounds did not. Since scrambled images lose the semantic information of the original images, we propose that semantic information produces the increase in hit rates. Some studies of scene perception have reported that semantic information of scene images is processed rapidly (see Thorpe, 2009 as a review) and automatically (for example, Li et al., 2002). Therefore, rapid and automatic processing of semantic information associates a target object in a short time, which suggests that the same semantic information in the background can work as a retrieval cue for the old probes. However, the facilitation effect was not observed on the false alarm rates, and meaningless backgrounds, such as scrambled ones, did not seem to produce the effect. A study of LTM by Rutherford (2004) observed a weak context effect similar to the one in the current study, demonstrating an increase in only the hit rates, which he explained by the cue-overload theory (Watkins and Watkins, 1975). This theory argues that the efficacy of contextual cues depends on the number of associations between targets and cues. It states that the fewer the number of targets assigned per cue, the more effective the cue becomes. Therefore, the cue-overload theory predicts greater hit rates or even greater recognition performances with the same rather than different contexts, when the number of cues is sufficiently large and the load per cue is low as, under such condition, it forms a sufficient number of distinguishable associations between targets and cues. In fact, Rutherford (2004) used three colors for backgrounds in word recognition tasks (assigning 20 words per color cue) and observed greater hit rates when the contexts were the same in the encoding and test phases, but did not observe the same context effect on the false-alarm rates. Conversely, when only one color was used as a background (60 words assigned per color stimulus), there was no difference in the hit and false alarm rates between the same and different contexts conditions. Isarida et al. (2005) also reported that higher hit rates under the same context condition when four colors were used as contexts (also assigning 20 words to each color cue), but did not find a context effect on false alarm rates. They also reported, however, that when twelve colors were used as contexts (6 words per a cue), not only hit rates, but also memory accuracy, increased. In the current study, although we assigned a single background image to a target, it is possible that the backgrounds have insufficient distinctiveness and, thus, the substantive number of cues was too small to cause complete cue-overloading, as categorically similar backgrounds were not likely to be discriminated clearly and they would have been assimilated. This would explain the facilitation effect of the same background scene; the scrambled backgrounds may have a subtle cue effect. Although it was not statistically significant, the hit rates in the Same and Different conditions seemed to be similar, and both of them seemed to be higher than in the NB condition. In addition, our participants gave us feedback after the experiment that they felt the scrambled images were less distinct. From the tendency of the results and the feedback from participants, we propose that the visual array information of our background images was not sufficiently distinct to serve as memory cues to improve the memory performance. Regarding scenes, Melcher and Murphy (2011) suggest that visual details of scene images tend to degrade quickly and only the coarse spatial layout of visual array information from the background images remains in memory. In addition, since Oliva and her collaborators indicated scene recognition involves coarse spatial layout (Oliva and Schyns, 1997; Oliva and Torralba, 2006), it seems reasonable that participants implicitly encoded the coarse layout of visual array information of scenes. This idea is further supported by the findings of a study by Torralba (2009), which reported that coarse scene images (at a low resolution) were not so distinct that participants confounded perceptually similar images (for example, low-resolution images that belong to the categories “highway” and “seaport” were incorrectly classified as belonging to the “beach” category). Further studies need to address the issue of distinctiveness for a memory cue.

The second conclusion from the results of our study is that changes in contexts disturb the retrieval of target objects. Both with scene and scrambled backgrounds, the backgrounds different from encoding made slower responses when old probes were presented. However, we assume the effect of changes in contexts involves two different aspects. Concerning scrambled backgrounds, hit RT under the NB condition was longer than under the Same or Different condition. Due to the lower distinctiveness of the scrambled images, participants may have regarded the white background in the NB condition as a “novel” background. This is similar to the result found by Hollingworth (2006, 2007). He suggested the association between a target object and a background context facilitates memory performance. It is possible that the absence of the associated context delays the retrieval of the associated object. Thus, the slower responses under the NB condition should be interpreted as another part of a facilitative context effect. In contrast, regarding scene backgrounds, correct rejection RT under the Different condition was longer than that under the NB condition. Since the association between an object and a background is novel in the correct rejection trials, the explanation by the association is not applicable to the correct rejection RT. We noticed the RT was longer when the scene context different from encoding was presented in recognition phase than when no context was given in recognition. Such effect, however, was not observed with scrambled contexts. This observation implies semantic information was involved in the increase of RT. It has been reported that the semantic information of scene images is processed automatically (Li et al., 2002), so that the processing of a novel scene is likely to occur during its presentation. We suggest that the slower responses in the correct rejection trials reflect the automatic processing the novel scenes and the formation of a new association between the scene and the object. Thus, we propose that a change in the semantic information of the scene context causes a disturbance effect.

In summary, the butcher-on-the-bus phenomenon in working memory consists of two different context effects in recognition: (1) a subtle facilitation effect by the same semantic information of contexts as in the encoding phases, and (2) a disturbance effect caused by changes of visual array information in contexts from the encoding phases. We presume the episode of “butcher-on-the-bus” reflect the latter disturbance effect. However, the scene context effects cannot be explained in a single uniform way, but should be considered as a combination of some processes. So far, studies of context effects have not differentiated the causes of these two effects; the apparent accuracy of recognition may reflect the context effects similar to our A’ result. In the conventional method to examine recognition memory, performances of both old and novel probes have been analyzed together, so the facilitation effect by old contexts might have been offset by the disturbance effect by novel contexts. Future research on context effects needs to distinguish between these two different effects to clarify the precise mechanisms of the influences of context information on memory. Conversely, it is conceivable that more highly developed cognitive abilities facilitate the complex processes of object memory with scene contexts. Unless we have abilities of modulation, processing task-irrelevant information will take a long time and we will fail to achieve the task goal. For example, people may fail to recognize information that they were instructed to remember because they process irrelevant context information. Our previous study reported that people occasionally recognized a part of the context by mistaking the information to be memorized, and suggested the tendency to avoid remembering irrelevant information reflects the ability of the central executive in working memory. In the current experiment, the context information is irrelevant to the task goal. Thus, context information should be inhibited and the contextual effects might be decreased. Actually, in the current study, we found that the context did not influence the index for memory accuracy (A’). However, other indices, such as hit rates and RTs, indicated two context effects (as described above) in the recognition of target information in backgrounds. It is arguable from these results that task-irrelevant information of context is processed, but most of it is inhibited by the working memory system before the memory judgment. Our present study indicates that future research needs to identify how working memory controls individual cognitive processes separately to understand visual working memory in the rich contexts of our daily life.

## Conflict of Interest Statement

The authors declare that the research was conducted in the absence of any commercial or financial relationships that could be construed as a potential conflict of interest.
